# Steroid hormonal endpoints in goats carrying single or twin fetuses reared in semi-extensive systems

**DOI:** 10.5194/aab-64-467-2021

**Published:** 2021-12-09

**Authors:** Luigi Liotta, Arianna Bionda, Deborah La Fauci, Marco Quartuccio, Rosanna Visalli, Esterina Fazio

**Affiliations:** 1 Department of Veterinary Sciences, Messina University, Viale Palatucci, 13, 98168, Messina, Italy; 2 BIOGENE, Veterinary diagnostic center, Via Giacomo Leopardi, 50, 95127, Catania, Italy

## Abstract

The study provides baseline data regarding 17- β -estradiol (E ${}_{2}$ ), progesterone (P ${}_{4}$ ), and cortisol profile of 30 Nicastrese goats during different physiological periods. Animals were evaluated monthly from the pre-mating period (non-pregnant), during pregnancy, and from 30 to 105 d of lactation. The effects of single or twin births and the kid's sex were also considered. Serum E ${}_{2}$ , P ${}_{4}$ , and cortisol concentrations were measured using immunoenzymatic assay kits. The highest concentrations of E ${}_{2}$ and P ${}_{4}$ ( P<0.0001 ) were found during pregnancy and their lowest values ( P<0.0001 ) in the non-pregnant period. E ${}_{2}$ was negatively correlated with P ${}_{4}$ ( r=-0.41 ; P<0.01 ) during lactation. The mothers with twin kids showed the highest concentration of P ${}_{4}$ ( P<0.04 ) at > 95–115 d of gestation and the lowest of E ${}_{2}$ ( P<0.04 ) at > 50–70 d of lactation. Pregnant goats carrying male kid(s) presented the highest E ${}_{2}$ concentrations ( P<0.02 ) at > 130–150 d of gestation. Different physiological conditions induced a temporal relationship with the endocrine profile in Nicastrese goats. Understanding the effects of single or twin fetuses on the gestation and lactation will also be helpful to improve the managemental approach for the health of mothers and their kids.

## 1 Introduction

Physiological gestation and lactation periods, especially the transition phase, are characterized by several metabolic and neuroendocrine changes intending to dynamically guarantee the growth of fetus and mammary glands (Iriadam, 2007; Krajničáková et al., 2004; Skotnicka, 2003). The interaction between the maternal–fetal unit implies an expression of greater metabolic stress in the mother with the advance of pregnancy approaching parturition, represented by high cortisol concentrations at parturition consequently to an increased fetal adrenal responsiveness to adrenocorticotropic stimuli (Ford et al., 1998); this event is correlated with a consistent and dynamic mammary growth and the milking start (Kitts, 1985). In goats, circulating cortisol concentrations are high during parturition and peak with the expulsion of the first kid; moreover, the observation of greater cortisol concentrations in goats with dystocia and retrained placenta suggests their higher stress level (Hydbring et al., 1999; Probo et al., 2011). Circumstantial evidence also suggests that cortisol helps the conversion of progesterone (P ${}_{4}$ ) into estrogen at the time of the delivery (Suganya et al., 2000).

17- β -Estradiol (E ${}_{2}$ ) concentrations in goats gradually increase with the advance of pregnancy (de Souza Castagnino et al., 2015), reach their maximum values at parturition, and consistently decrease on the following day (Capezzuto et al., 2008; Probo et al., 2011). An opposite trend was shown for P ${}_{4}$ , with a prepartum decline correlated to the onset of parturition (Laura et al., 2004) or around kidding (Capezzuto et al., 2008; Probo et al., 2011).

The corpus luteum is the only source of P ${}_{4}$ for the physiological maintenance of pregnancy in goats and an increased number of corpora lutea may induce a greater P ${}_{4}$ and E ${}_{2}$ secretion, leading to the stimulation of mammary gland growth in goats carrying twin fetuses (Khan and Ludri, 2002b). Nevertheless, in Surti goats no significantly higher E ${}_{2}$ values were observed in twin gestations than single-bearing mothers (Gamit et al., 2019). On the other hand, a low level of E ${}_{2}$ was recorded in twin compared to single pregnancy in crossbred goats by Khan and Ludri (2002a). The simultaneous changes in maternal circulating E ${}_{2}$ , P ${}_{4}$ , and cortisol concentrations during the periparturient period in goats carrying single and twin fetuses were also observed, with the highest P ${}_{4}$ values in twin-bearing goats (Khan and Ludri, 2002b; de Souza Castagnino et al., 2015) and the highest E ${}_{2}$ and cortisol values on the day of kidding in both single and twin pregnancies (Khan and Ludri, 2002b).

The effects of gestation and post-kidding stages on hematochemical and hormonal parameters in goats were investigated well (Cepeda-Palacios et al., 2018; Hefnawy et al., 2011; Khan and Ludri, 2002a, c; Madan et al., 2020; de Souza Castagnino et al., 2015); nevertheless, a perusal of the literature indicates that few comprehensive studies have been carried out on the profile of steroid hormones throughout the whole gestation, peripartum, and postpartum periods in different breeds of dairy goats carrying single or twin fetuses (de Souza Castagnino et al., 2015). Furthermore, the same comparative hormonal endpoints were often recorded on ewes, but these data might be not comparable with those of the goat species. Information regarding this topic would be desirable, since the physiological reproductive performance of goats plays an important role in improving the milk and meat sustainability, given their specific ability to cope under unfavorable environmental conditions (Devendra, 2001; Tharwat et al., 2013). The hypothesis was that the major changes in the maternal endocrine response were caused by the progression of pregnancy, regardless of the number of fetuses.

The largest number of regional goat breeds are found in Italy (http://www.assonapa.com, last access: 20 October 2021). In this study, we evaluated the Nicastrese goat, a local breed reared in the Calabria region, a southern Italian region in which about 4800 individuals can be found, reared in 77 flocks. The Calabrian goat farming system mainly consists of small- to large-sized farms, spanning free-ranging to semi-sedentary and natural pasture-based management (Usai et al., 2006). The typical aromatic Mediterranean plants present in the natural grazing lands of this area confer typical sensorial features to the milk and the Nicastrese cheese (De Nardo, 2014; Nicoloso et al., 2015; Pino et al., 2018; Randazzo et al., 2014). Nicastrese goats show a black coat with the limbs, abdomen, and part of the head white, a straight frontal–nasal profile, and a typical head with lyre-shaped horns in both sexes. Adult males and females have a body weight of about 78 and 46 kg, respectively. Primiparas show a lactation of 180 d of duration and with a milk production of 150 kg; multiparous goats show a lactation that lasts from 220 to 260 d, with a milk production of about 210 kg. Milk has an average fat content of 4.30 %, a protein content of 3.50 %, and a lactose content of 4.70 % (Pino et al., 2021). The aim of the present study was to investigate E ${}_{2}$ , P ${}_{4}$ , and cortisol changes in non-pregnant, pregnant, and lactating Nicastrese goats, by considering the effect of single or twin fetuses and, related to this, the kid's sex. In doing this, we wish to generate baseline data of the endocrine profile of native goat breeds usually reared in semi-extensive conditions.

## 2 Materials and methods

The experimental protocol was approved by the Ethical committee of the Department of Veterinary Science of the University of Messina, Italy (code 046/2020).

The research complied with guidelines of Good Clinical Practices (EMEA, 2000). This study was performed according to the Italian and European regulations on animal welfare (Directive 2010/63/EU of the European Parliament and of the Council of 22 September 2010 on the protection of animals used for scientific purposes).

### 2.1 Animals and breeding

Thirty multiparous Nicastrese goats, aged from 3 to 4 years, were considered during the transition period (from March 2020 to January 2021). Animals were randomly sampled from a flock of 400 goats, stabled using semi-extensive farming management in a commercial farm located in Catanzaro (39.048 ${}^{\circ }$ N, 16.5653 ${}^{\circ }$ E; 1215 m above the sea level), a province of Calabria: this farm was chosen because, rearing almost 10 % of the total Nicastrese goat population (composed of about 4800 animals, as previously reported) in homogeneous conditions, it could be regarded as representative of the breed. All the goats grazed in the same pasture area, mainly consisting of natural woodland pasture, from 08:00 to 16:00 CEST, whereas they were confined in a stable covered with straw bedding during the night. There, all the animals, independently of their physiological status, were fed with concentrate (on average 0.7 kg per head per day; crude protein: 155.1 g kg ${}^{-1}$ of dry matter (DM); ether extract: 41.2 g kg ${}^{-1}$ of DM; neutral detergent fiber: 196.1 g kg ${}^{-1}$ of DM; net energy used for lactation: 1.08 UFL (Forage Unit for Lactation) kg ${}^{-1}$ of DM) and meadow hay (on average 1 kg per head per day; crude protein: 110.9 g kg ${}^{-1}$ of DM; ether extract: 25.0 g kg ${}^{-1}$ of DM; neutral detergent fiber: 521.9 g kg ${}^{-1}$ of DM; net energy used for lactation: 0.65 UFL kg ${}^{-1}$ of DM); water was available ad libitum.

All goats had normal estrus cycles and were free of uterine disease. Goats became pregnant between the second half of June and the beginning of July; the first week after the seasonal mating was regarded as the first week of gestation. All animals reported normal pregnancy and spontaneous, eutocic delivery. Parturition occurred in 30 goats after a mean gestational length of 150 d (145–155 d); animals were divided into two groups: 13 goats delivered a single kid and 17 goats delivered twin kids. All kids were alive, healthy, and diagnosed as clinically normal at the postpartum veterinary examination. The kids were fed on their dam's milk until weaning.

All samples were taken between 07:00 and 09:00 in the morning to minimize the effect of the circadian rhythm on hormone measurements, in quiet conditions by the same operator.

### 2.2 Samples and analyses

Blood samples were collected from the external jugular venipuncture into tubes containing clot activator and separating gel (Terumo Corporation, Tokyo, Japan). Samples were centrifuged at 1300 g for 10 min, and the obtained serum samples were aliquoted and stored at 4 ${}^{\circ }$ C until the analyses, which were performed within 1 week at the Veterinary Diagnostic Center BIOGENE (Catania, Italy).

Serum E ${}_{2}$ , P ${}_{4}$ , and cortisol concentrations were assayed using a homologous solid-phase, two-site chemiluminescent immunometric assay (Immulite^®^ 2000, Siemens Medical Diagnostic Solutions, USA), according to the manufacturer's instructions. E ${}_{2}$ intra- and inter-assays coefficients of variation (CVs) were 2.2 % and 5.1 %, respectively. The sensitivity of the assay was 9 pg mL ${}^{-1}$ . P ${}_{4}$ intra- and inter-assays CVs were 5.7 % and 3.8 %, respectively. The sensitivity of the assay was 0.25 ng mL ${}^{-1}$ . Cortisol intra- and inter-assays CVs were 0.27 % and 6.1, respectively. The sensitivity of the assay was 0.20 µ g dL ${}^{-1}$ .

### 2.3 Statistical analysis

The software JMP^®^ 15 (SAS Institute Inc., Cary, NC, USA) was used to perform the statistical analyses. The parameters and results were described as mean ± standard deviation of the mean. A mixed model was used to compare all the examined parameters among the different physiological phases (pregnancy, lactation, and non-pregnancy) and the different months within each phase, as well as for single vs. twin pregnancy and for the kids' sex (male, female, and male + female); animals were regarded as random effect in order to account for the correlation between the repeated measurements within each subject (Detry and Ma, 2016). Tukey's HSD (honestly significant difference) post hoc test allowed us to discriminate between the differences between the groups. The correlation between the parameters was assessed with Pearson's correlation coefficients ( r ). The conventional threshold of 0.05 was used to determine the significance of the results.

## 3 Results

**Figure 1:**
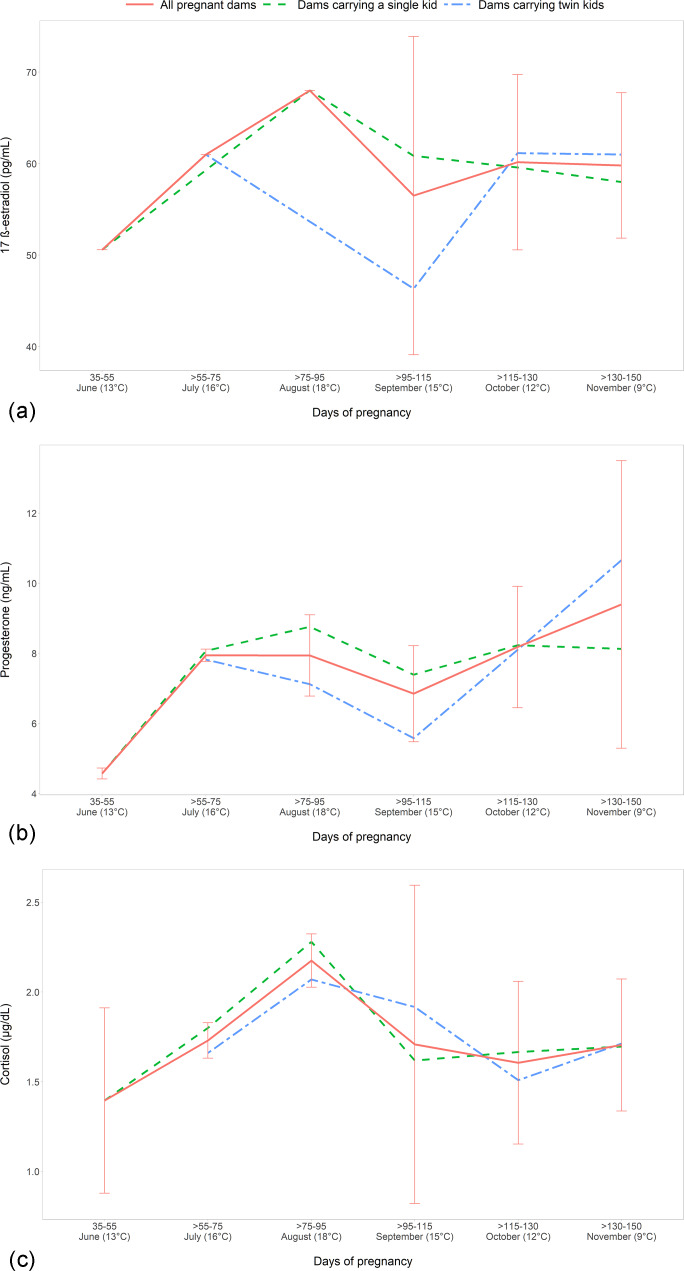
Circulating 17- β -estradiol **(a)**, progesterone **(b)**, and cortisol **(c)** concentrations (mean ± SD) in 30 pregnant Nicastrese goats.

**Figure 2:**
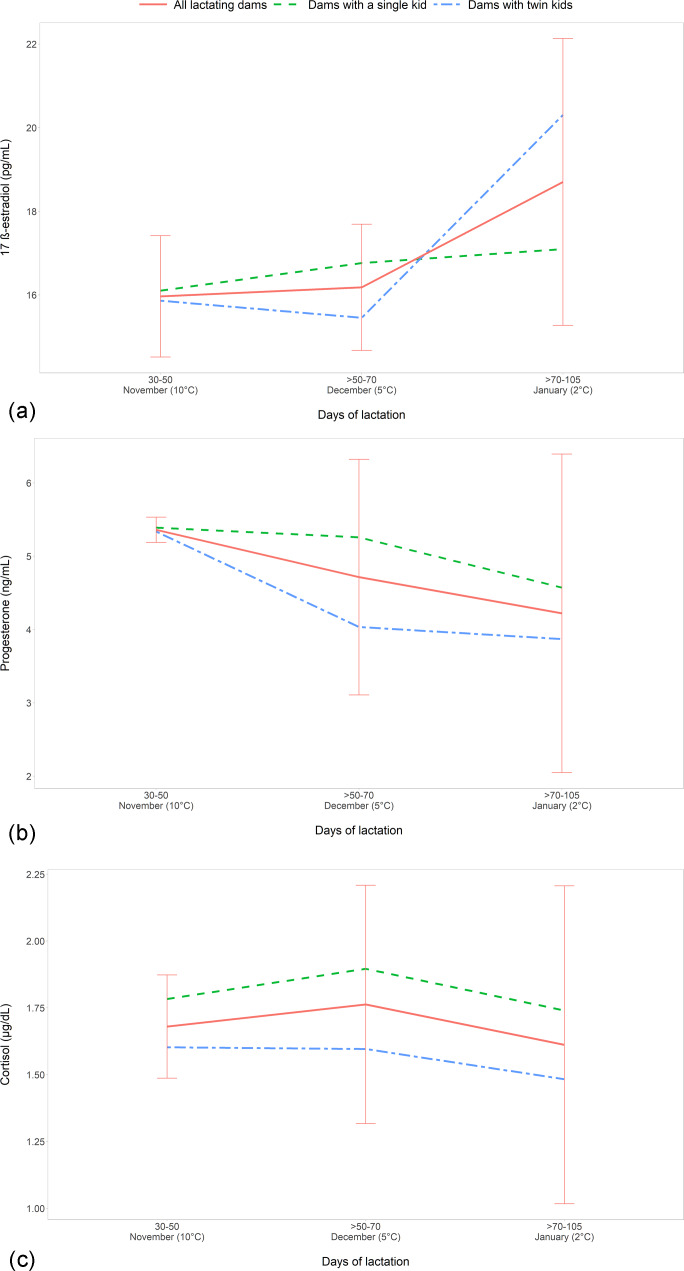
Circulating 17- β -estradiol **(a)**, progesterone **(b)**, and cortisol **(c)** concentrations (mean ± SD) in 30 lactating Nicastrese goats.

The mean ± SD and the range of circulating E ${}_{2}$ , P ${}_{4}$ and cortisol concentrations measured during different physiological phases (non-pregnancy, pregnancy, and lactation) are shown in Tables 1–2 and Figs. 1–2.

The comparison among the different physiological periods (Table 1) showed the highest E ${}_{2}$ and P ${}_{4}$ concentrations in pregnant goats and their lowest values in the non-pregnant period ( P<0.0001 ). No significant differences were observed for cortisol changes.

**Table 1:** Circulating 17- β -estradiol, progesterone, and cortisol concentrations (mean ± SD) and minimum and maximum values in different physiological periods of 30 Nicastrese goats. Within each row, different superscript letters indicate significantly different values.

Periods	Non-pregnancy	Pregnancy	Lactation	P value
17- β -Estradiol (pg mL ${}^{-1}$ )	21.65 ± 9.55 ${}^{B}$	59.10 ± 11.64 ${}^{A}$	16.96 ± 2.56 ${}^{B}$	<0.0001
	14.17–33.59	15.00–69.00	14.22–25.00	
Progesterone (ng mL ${}^{-1}$ )	0.59 ± 0.35 ${}^{C}$	8.27 ± 2.96 ${}^{A}$	4.69 ± 1.66 ${}^{B}$	<0.0001
	0.30–1.43	0.56–16.92	0.25–5.67	
Cortisol ( µ g dL ${}^{-1}$ )	1.57 ± 0.58	1.68 ± 0.51	1.70 ± 0.46	0.7521
	1.02–2.91	1.01–3.34	1.05–3.19	

The evaluation of the non-pregnant goats' hormonal pattern between March and May (Table 2) showed that E ${}_{2}$ concentrations were higher ( P<0.002 ) in April, without significant differences for cortisol and P ${}_{4}$ values.

**Table 2:** Seasonal circulating 17- β -estradiol, progesterone, and cortisol concentrations (mean ± SD) in 30 non-pregnant Nicastrese goats.

Months	March	April	May	P value
Temperature	5 ${}^{\circ }$ C	7 ${}^{\circ }$ C	9 ${}^{\circ }$ C	
17- β -Estradiol (pg mL ${}^{-1}$ )	14.72 ± 0.48	32.04 ± 2.20	/	0.0025
Progesterone (ng mL ${}^{-1}$ )	0.71 ± 0.62	0.35 ± 0.04	0.61 ± 0.21	0.6757
Cortisol ( µ g dL ${}^{-1}$ )	1.25 ± 0.70	1.97 ± 1.34	1.60 ± 0.40	0.5050

When the different months of pregnancy, ranging from June to November, were compared, no significant differences were found, but we observed that E ${}_{2}$ , P ${}_{4}$ , and cortisol (Fig. 1a, b, and c, respectively) gradually increased with the advance of the early pregnancy (from days 35 to 95) and then decreased (until the 135 d for cortisol and until the 115 d for E ${}_{2}$ and P ${}_{4}$ ), with a final increase for all hormones until 150 d. The lowest concentrations of all the investigated hormones were observed in June, between 35 and 55 d of gestation. The highest values of E ${}_{2}$ and cortisol were measured in August, from 75 to 95 d of gestation, and the highest P ${}_{4}$ values in November, at the end of the pregnancy.

The comparison among the different periods of lactation, ranging from 30–50 to > 70–90 d postpartum (Fig. 2), showed a gradually significant E ${}_{2}$ increase ( P<0.01 ) at > 70–90 d, with the advance of lactation. A significant and negative correlation between E ${}_{2}$ and P ${}_{4}$ values ( r=-0.41 ; P<0.01 ) was observed during the lactation period.

The evaluation of the effect of single or twin fetuses during the whole pregnancy showed that the mothers with twin fetuses had generally the highest E ${}_{2}$ and P ${}_{4}$ concentrations (Fig. 1a and b, respectively) in the first part of pregnancy and the lowest values after the 115th day, with the significant highest P ${}_{4}$ concentrations at > 95–115 d ( P<0.04 ).

Regarding the sex of the kid, the mothers with single or twin male kids showed the highest E ${}_{2}$ values ( P<0.02 ) at > 130–150 d of gestation.

The evaluation of the effect of single or twin fetuses during lactation showed that the mothers with twin kids had the lowest P ${}_{4}$ and cortisol (Fig. 2b and c, respectively) values in all time points of lactation, whereas E ${}_{2}$ (Fig. 2a) concentration had significantly lower results ( P<0.04 ) at > 50–70 d, with a tendency to increase at > 70–105 d.

## 4 Discussion

The overall mean E ${}_{2}$ , P ${}_{4}$ , and cortisol ranges recorded in this study are in accordance with the physiological ranges reported by different authors for clinically healthy non-pregnant, pregnant, and postpartum goats (Gamit et al., 2019; Khan and Ludri, 2002c; Probo et al., 2011; de Souza Castagnino et al., 2015; Tharwat et al., 2013). Nevertheless, some variation might be due to differences in the techniques used (Barth et al., 2018), the experimental model, diet, management, the physiological period, and genotype. Moreover, the data obtained exclude the possible influence of circadian rhythms because the blood sampling was always performed at the same time of the day, between 07:00 and 09:00, in non-pregnant, pregnant, and postpartum periods.

In the present study, we found the significantly highest values of both E ${}_{2}$ and P ${}_{4}$ in the whole pregnancy compared with the prior and following periods, consistently with an increased demand for a higher metabolic rate for pregnancy, previously observed in dairy goats (de Souza Castagnino et al., 2015). This trend might happen due to the preparation of the reproductive system for the growth of the uterus and fetus, as previously recorded for goats (Capezzuto et al., 2008; Gamit et al., 2019; Laura et al., 2004). On the other hand, the significant decrease in E ${}_{2}$ and P ${}_{4}$ in early lactation may also reflect a difference in nutrient availability and the related utilization in a catabolic or anabolic manner: this may temporarily change the metabolic requirements to cope with an intensive lactogenesis phase. Sure enough, goats showed a peak of lactation around 40 d, with a depressed feed intake, primarily of dry matter, at the onset of lactation (catabolic phase) and then an increase in its ingestion (anabolic phase). Therefore, the reduction in ovarian activity observed in lactating goats may reflect a seasonal reduction in fertility, possibly linked with increasing temperature and photoperiod (Llewelyn et al., 1995).

It should also be considered that during lactation the hormonal concentration may also be affected by the hemodilution resulting from a physiologically increasing water metabolism to mammary glands through the vascular system, as previously observed for hematological and biochemical compounds in lactating ewes (Brito et al., 2006). It can also not be excluded that the significantly lowest E ${}_{2}$ and P ${}_{4}$ concentrations during the whole lactating period considered, with a special emphasis on 30–50 d postpartum, are suggestive of no initiation of ovary activity, as corroborated by the negative and significative correlation between P ${}_{4}$ and E ${}_{2}$ observed only in lactating goats. On the basis of these first considerations, it is possible to presume that the transition period represents the most stressful time in the production cycle of dairy goats, like previously recorded in periparturient goats (Tharwat et al., 2013) and dairy cows (Guo et al., 2007).

Although no significant differences were present among the different pregnancy months, a gradual increase in E ${}_{2}$ and P ${}_{4}$ concentration was observed as the pregnancy progressed. This same trend was found in the study of de Souza Castagnino et al. (2015), in accordance with the knowledge that the corpus luteum is the only source of P ${}_{4}$ for the physiological maintenance of pregnancy in the goat (Khan and Ludri, 2002b); in addition, the trend of E ${}_{2}$ represented the physiological inhibitory mechanism that prevents the maturation of other follicles.

During pregnancy, cortisol concentrations showed a similar trend to E ${}_{2}$ and P ${}_{4}$ , increasing in the first months and then decreasing. This could be attributed to an increased hepatic glycogenolysis and mobilization of amino acids for gluconeogenesis, more active during the first trimester. Moreover, the cortisol secretion by the adrenocortex is essential for the induction of several gluconeogenic enzymes that enable the animal to cope with stressful conditions, as soon as the morpho-functional changes occur during the whole pregnancy. It is well known that, during pregnancy, fetuses have a large glucose demand that is satisfied by the mother and that about 60 % of ovine fetal growth takes place in the last 4 to 6 weeks of gestation (Hefnawy et al., 2011). Thus, after the first trimester (from >95 d), pregnant goats may activate a glucocorticoid inhibitory mechanism as a response of the hypothalamic–pituitary–adrenal axis to stress, in order to prevent blood glucocorticoid concentrations becoming too high, according to the fetus' demand. The resemblance of E ${}_{2}$ and cortisol trend would confirm that the estrogen reduces the metabolic clearance rate of cortisol (Kitts, 1985).

Although no significant differences among 35–150 d of pregnancy for cortisol, E ${}_{2}$ , and P ${}_{4}$ were recorded, the data obtained reported the highest E ${}_{2}$ and cortisol values in August, from 75 to 95 d, and the lowest values in June, from 35 to 55 d of pregnancy. These seasonal changes support the knowledge that, compared to other domestic animals, goats show a good adaptive plasticity, depending upon to the genotype, which allows them to anticipate climate change, thus adapting their physiology, specifically that of reproduction (Farsi et al., 2018). Likewise, the effect of temperature variation on hormonal concentration at various gestation stages in Black Bengal goats was also recorded, showing that a rise in temperature has no deleterious effect on the metabolic or the reproductive hormonal concentrations (Kumar et al., 2015). In addition, it is plausible that there was a large amount of individual variation in the animals during different physiological periods, which could be one additional reason why no significant differences were found.

Related to the effect of single or twin fetuses on the hormonal endpoints of mothers, the highest E ${}_{2}$ and P ${}_{4}$ concentrations observed in goats carrying twin fetuses also confirm that an increased number of corpora lutea may induce a greater P ${}_{4}$ and E ${}_{2}$ secretion, leading to the stimulation of mammary gland growth, as previously described in twin-fetuses-bearing crossbred goats (Khan and Ludri, 2002b), in Surti goats (Gamit et al., 2019), in dairy goats (de Souza Castagnino et al., 2015), and in does during late pregnancy (Manalu et al., 1997). Particularly, in twin-bearing goats, E ${}_{2}$ and P ${}_{4}$ concentrations were higher from the first stage of pregnancy until 115 d and lower in the last period from >115 to 150 d. These results are in accordance with the hormonal profile described in single- and twin-bearing Beetal goats during the transition period, both at -15 d, and only for P ${}_{4}$ at day 30 postpartum (Madan et al., 2020).

The determination of the effect of the kid's sex on the hormonal changes in the mother has not been previously reported for goats; nevertheless, previous results showed that male only or male and female fetuses induced significant maternal cortisol increases in postpartum crossbred ewes carrying single or twin fetuses (Fazio et al., 2013). In the present study no significant differences were observed nor for the cortisol or for P ${}_{4}$ changes in mothers; nevertheless, E ${}_{2}$ concentrations were higher in mothers with single or twin male fetuses, showing that the sex of fetus could redirect the placental synthesis of steroid hormones from progesterone towards estrogens, leading to increased values in the final period of pregnancy, according to the evidence speculated on by Ford et al. (1998) on the endocrine changes approaching parturition in Angora-cross goats.

From the study, it can be considered that, under similar environmental conditions, nutritional regime, and management systems, the physiological status plays a significant role in E ${}_{2}$ , P ${}_{4}$ , and cortisol secretion, affecting dynamically the steroid endocrine pattern in Nicastrese goats.

The obtained physiological values may be used as additional resources to improve desirable observations, especially when the different physiological conditions are taken into account. Doing this, we wish to generate baseline data of the endocrine profile of a native goat breed usually reared in semi-extensive conditions.

Likewise, with the constant pressure of the small-ruminant industry to develop and optimize methods to promote the production of milk derivatives, it appears that continuing research on this topic remains kindly encouraged.

## 5 Conclusions

In this study, we consolidate the small amount of previously reported physiological data with an additional result that reflects a interaction between hormonal patterns and functional periods. These data contribute to the overall knowledge on the physiological 17- β -estradiol, progesterone, and cortisol patterns of Nicastrese goats, mainly intended for milk production for local cheese manufacturing. Understanding the effects of twinning during the transition period will be helpful to improve the lack of new techniques and improper management skills. In conclusion, these original data can help Calabrian farmers and others in the rescue of this endangered, local, native breed. Growing and completing the development of the young stocks of goats on their farms of origin as well as promoting Nicastrese milk and cheese as “local niche” food could, in fact, increase farmers' incomes. Moreover, the husbandry of this autochthonous goat breed contributes to the maintenance of the fragile Calabrian environment. If a conservation program is undertaken, rare specimens and their genetic variability will be preserved. Thus, our results aim to encourage further research on this issue.

## Data Availability

The datasets generated and/or analyzed during the current study are available from the corresponding author on reasonable request.
